# Rodent Seed Dispersal Syndromes Follow a Downslope Trajectory, Counteracting the Climate Change‐Mediated Tree Line Elevational Shift Upwards

**DOI:** 10.1002/ece3.71388

**Published:** 2025-05-12

**Authors:** Ning Han, Jing Wang, Tuo Feng, Jidong Zhao, Jianghong Zhang, Xiang Hou, Gang Chang

**Affiliations:** ^1^ Shaanxi Key Laboratory of Qinling Ecological Security Shaanxi Institute of Zoology Xi'an China; ^2^ Zhenba County Farmers' Science and Technology Education Training Center Hanzhong China

**Keywords:** plant dispersal, rodents, seed caching, slope effects, terrain

## Abstract

Forest rodents are important mediators of plant seed dispersal and their seed caching tactics are influenced by a variety of environmental factors; however, the role of terrain slope remains uninvestigated. We examined how the dispersal of *Castanea mollissima* seeds by an assemblage of scatter‐hoarding rodents in the Qinling Mountains, China, was affected by slope direction and gradient in relation to seed fate. In this study, the topographic factor, which has been frequently overlooked in previous ecological studies, was investigated. It was revealed that the sloping terrain could affect the dispersal behavior of rodents toward plant seeds and ultimately influence the direction of plant dispersal. This finding brings new insights to ecological research. Overall, rodents were 1.55 more likely to transport seed downhill than uphill, and downhill mean translocation distance was 1.41 times greater than uphill, suggesting an overarching tendency for energy conservation. When comparing steep (> 35°) with shallow (< 35°) slopes, this gradient effect was strongest on gentle slopes, with other factors likely exerting a greater influence on steeper terrain. We discuss these findings both from the perspective of rodent optimal foraging in ‘landscapes of fear’ and heterogeneous ‘energy landscapes’, as well as in the context of the counteractive pressure for trees to achieve an uphill elevational shift in response to global warming.

## Graphical abstract

In the Qinling Mountains, China, the impact of terrain slope on the dispersal of *Castanea mollissima* seeds by forest rodents was studied. It was found that rodents were more likely to transport seeds downhill and over a greater distance, with the gradient effect being strongest on gentle slopes. This study reveals the influence of the often‐overlooked topographic factor on rodent seed dispersal behavior and plant dispersal direction, bringing new insights to ecological research.

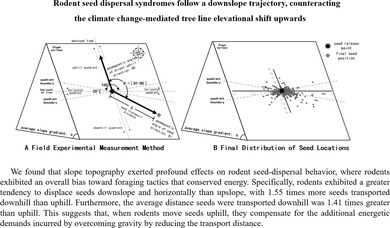

## 1 Introduction

Rodent‐mediated seed dispersal plays a vital role in ecosystem functionality and the persistence of biodiversity (Vander Wall 1990). Seeds transported to favorable new sites, and thus avoiding proximity‐ and density‐dependent mortality, result in plant recruitment success and the colonization of new areas (Vander Wall 1990). In turn, seeds provide rodents with an important food source, and thus a proportion of all seeds produced by plants are lost to seed predation (Chang and Zhang 2014; Fedriani and Manzaneda 2005; Moore et al. 2022) or caching for later consumption (Bogdziewicz et al. 2019).

In order to avoid the loss of stored food to decay or intra‐guild competitors (Cao et al. 2018), rodents select secure microsites to cache food (Wang and Corlett 2017), such as in rock fissures, dense ground vegetation, or buried in soil (Bogdziewicz et al. 2019). If safe caching microsites were randomly distributed across the landscape, rodents would likely transport seeds to these sites in random directions, resulting in non‐directional seed dispersal patterns around the parent tree (Shuai and Song 2011; Zhang et al. 2008). In reality, however, seed dispersing rodents generally perform directed dispersal (Briggs et al. 2009), influenced by seed type, seed handling and transportation energy costs, energy gain from consumption, and the long‐term utility of seeds for future consumption for example, nutritional value, resistance to decay; (Moore and Swihart 2008; Xiao et al. 2004, 2005).

Scatter‐hoarding rodents must balance energy expenditure during foraging, territorial defense, and other activities against energy rewards to maximize net fitness (Chimienti et al. 2020). For small rodents (e.g., *Apodemus draco* , a common scatter‐hoarding species in the study area, body mass ~15–25 g), a 7 g seed can constitute a substantial proportion of their own body weight, and therefore carrying this load over a distance exerts an energy expenditure cost (Moore et al. 2007). In montane habitats, uphill seed transport by rodents incurs higher energetic costs (e.g., working against gravity), whereas downslope transport is more energy‐efficient (Birn‐Jeffery and Higham 2014) According to optimal foraging (Lichti et al. 2017) and energy landscape theories (Shepard et al. 2013), rodents are more likely to prioritize downhill seed dispersal to minimize energy expenditure, thereby influencing the directional bias of seed movement. Rodents' energy‐saving strategy of preferring downhill seed dispersal may counteract the uphill migration of tree species driven by climate warming. However, as global temperatures rise, plant communities are forced to shift to higher elevations to remain within their adapted bioclimatic envelopes (Corlett and Westcott 2013; Kelly and Goulden 2008; Lenoir et al. 2008; Naoe et al. 2019). However, studies on vertical seed dispersal remain limited (González‐Varo et al. 2017; Naoe et al. 2019; Zhang et al. 2022). Therefore, understanding whether seed dispersal trajectories exhibit a general downhill bias—contrary to the adaptive need for many plant communities to migrate uphill under climate warming—is critical for predicting future forest community structures (Alexander et al. 2018; Frei et al. 2010; Ruiz‐Labourdette et al. 2011).

To address this knowledge gap, we hypothesized that rodents would preferentially disperse seeds downhill to minimize energy costs, thereby counteracting climate‐driven uphill tree migration. In this study, we investigated whether a montane rodent assemblage exhibited bias in its vertical seed dispersal, specifically analyzing seed dispersal trajectories and distances, and seed weight, in relation to ultimate seed fates. We then considered the implications of our findings in the context of optimal foraging theory and climate‐driven elevational shifts in forest plant communities.

## 2 Materials and Methods

### 2.1 Study Sites

This study was conducted in Shaanxi Foping National Nature Reserve between September 20nd to November 8th 2022 and September 21st to November 8th 2023. This is the natural period when local Fagaceae seeds fall and are transported and stored by rodents. The reserve is located on the southern slope of the central section of the Qinling Mountains (33°33′ N–33°46′ N, 107°41′ E–107°55′ E), comprising the northern edge of the transition zone between the northern subtropical and warm temperate climatic zones (average annual temperature: 11.4°C; average annual precipitation: 943 mm). The natural vegetation types are divided into three vertical natural zones by elevation height: conifer forest (above 2500 m), mixed broad‐leaved and conifer forest (between 2000 and 2500 m), and deciduous broad‐leaved forest (below 2000 m) (Chang et al. 2012). The study area is situated at an elevation of 1200 m in the deciduous broad‐leaved forest zone that typifies the lower and middle mountain habitats in this region. In this forest, Fagaceae constitute the dominant species, principally *Quercus aliena*, *Q. variabilis* , *Q. glandulifera*, and *Castanea mollissima* . This region also has a diverse rodent community, including *Niviventer confucianus* , *Apodemus draco* , *A. chevrieri* , *A. peninsulae* , *Cansumys canus* , *Mus minutoides* , and *Sciurotamias davidianus (*Wang et al. 2021, 2017).

### 2.2 Seed Collection and Marking

Each year, during the *C. mollissima* (Chinese chestnut) seed ripening period (late September), 500 intact *C. mollissima* seeds (i.e., chestnuts) were collected (Chen et al. 2017). Each seed was weighed and marked using plastic tags (2.5 cm ×3 cm ×0.01 cm; < 0.1 g) (Xiao et al. 2005). This tin/plastic‐tagged method does not affect rodents' seed dispersal and hoarding behaviors (Ho et al. 2021; Wang et al. 2021; Xiao et al. 2005), while enabling efficient and accurate tracking of individual seed fates through rapid tag identification.

### 2.3 Field Experiment Procedure

Ten experimental seed stations (seed release sites, minimum distance between sites 50 m, chosen to eliminate inter‐site interference and exceed rodent seed dispersal distances) were established on uniformly sloping terrain within the mountainous forest zone of the study area. All sites comprised similar plant communities and vegetation cover. For each seed release site, the bearing (D0) of maximum incline was measured using a compass. This bearing of maximum incline was taken as the zero‐degree angle at the release site, and the slope gradient located 1 m away from this point was measured using a gradient meter at eight equally divided azimuths (0°, 45°, 90°, 135°, 180°, 225°, 270°, 315°). The average of these measurements was then used to calculate the mean slope gradient α for each site and classified as gentle (*α* < 35°) or steep (*α* ≥ 35°).

**FIGURE 1:**
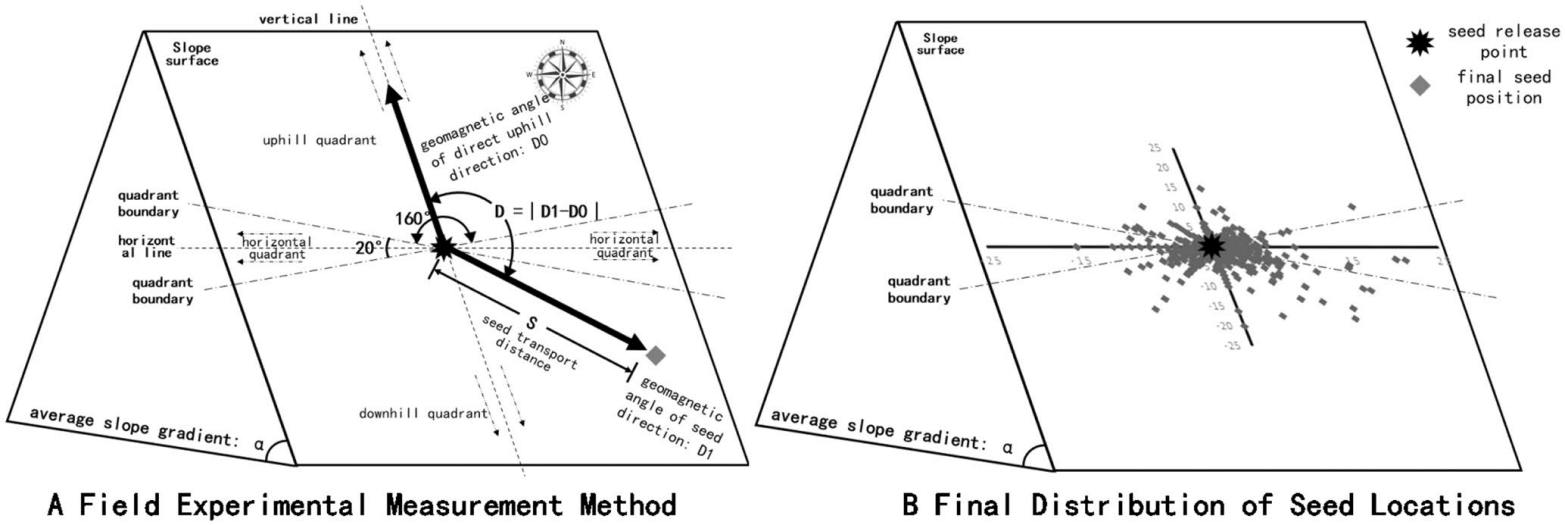
Schematic diagram of the field seed dispersal experiment.

In October 2022 and 2023, 50 uninfested chestnuts in good condition and of known weight were labeled and placed in a pile at each of the 10 selected release sites (seed station), and then tracked daily for 30 consecutive days. This is based on our previous experimental observations; this approach ensures that over 90% of the seeds are detected and processed by rodents. Seeds were tracked within a 30 m radius of each seed station, as rodent seed dispersal distances in montane forests typically do not exceed this range (Briggs et al. 2009; Chang et al. 2012; Moore et al. 2007; Xiao et al. 2005; Wang et al. 2017). The initial locations and distances (S) of seed dispersal were recorded (subsequent relocation of seeds was discarded), and the fates of all experimental seeds were recorded based on where the seed tags were relocated and categorized as: (1) Intact in situ (IIS): Seeds remained intact at the release point; (2) Eaten in situ (EIS): Seeds were consumed at the release point; (3) Consumed after Moving (CAM): Seeds were consumed after being moved; (4) Scatter hoarded after Moving (HAM): Seeds were scattered and buried in the soil or grass after being moved; (5) Larder hoarded after Moving (LHM): Seeds remained intact at centralized storage sites (typically rodent nests); (6) Discarded after moving (DAM): Seeds were discarded intact on the ground surface after being moved (Cui et al. 2018). As the objective of this study was to investigate the effect of mountain slope on seed dispersal, all IIS and EIS seeds were excluded from further analyses. Additionally, all LHM seeds were excluded as larder hoarding typically occurs in rodent burrows or sheltered microsites (e.g., rock crevices, root cavities), where cache location is primarily driven by safety rather than energy costs, which are central to this study's focus on slope effects. The bearing (D1) between the original seed release site and its initial dispersed location was measured. The angle D (D = ∣D1—D0∣) between this seed dispersal bearing and the maximum incline bearing was then calculated to determine the slope direction in which the seed was dispersed, classified as dispersal upslope (0° ≤ D < 80° or 280° < D < 360°), downslope (100° < D < 260°), or horizontally (i.e., flat) along the slope (80° ≤ D ≤ 100° or 260° ≤ D ≤ 280°) (Figure 1‐A). Finally, the number, percentage, weight, and dispersal distance were calculated for each individual seed released at each site and attributed to one of these three slope direction groups for statistical comparison.

### 2.4 Statistics and Analysis

Data analysis was performed using SPSS version 20.0. An exploratory analysis was conducted initially to ascertain that data followed a normal distribution. Subsequently, a General Linear Models‐Univariate (GLM‐U) analysis was employed to assess the main effects of various influencing factors such as seed weight, fate, seed dispersal distance, and slope gradient on seed dispersal distance, seed weight, and the proportion of seeds in each directional category (upslope/downslope/horizontal). A Chi‐square test was applied to analyze the percentage of dispersed seeds in the upslope, horizontal, and downslope displacement classes. Furthermore, the interaction of the slope gradient α with seed weight, fate, and seed dispersal distance in each slope direction category (horizontal, upslope, downslope dispersal) was examined using separate tests for each parameter. The level of significance was set at *p* = 0.05.

## 3 Results

From the 1000 chestnut seeds tracked over 2022 (*n* = 500) and 2023 (*n* = 500), we ascertained a total of 721 valid seed fates categorized by slope direction. Three seeds dispersed farther than 30 m were excluded from further analysis because this distance implies avian or rodent multiple dispersal, rather than rodent one‐time dispersal, along with the exclusion of 36 LHM seeds cached centrally in burrows, where burrow location rather than slope likely affected the dispersal directions of these seeds. This yielded 682 valid seed dispersal directions for analysis, including 499 HAM (73.2%), 148 CAM (21.7%), and 35 DAM seeds (5.1%). Among these, 352 seeds were transported to downhill locations from their release points (51.6%), 103 were moved to level positions relative to the release points (15.1%), and 227 were dispersed uphill from their original positions (33.3%, Table 1, Figure 1B).

**TABLE 1:** Statistical differences in seed dispersal trajectories.

Dispersal trajectory class	Number of seeds (*n*)	Percentage (%)	Average weight (g)	Average distance (m)
Downslope	352	51.6	7.37	4.37
Horizontal	103	15.1	7.06	3.66
Upslope	227	33.3	7.37	3.10
Total	682	100	7.32	3.84
*p*‐values	0.000000^a^	0.000000^a^	0.384	0.000086^a^
Wald Chi‐squared	36.78	36.78	0.95	9.32

^a^ Denotes highly significant.

The tendency for rodents to preferentially move seeds downslope was thus significant (*p* < 0.01, χ^2^ = 36.78). Furthermore, there was a significant tendency for longer distance downslope dispersal (*p* < 0.01, χ^2^ = 9.32) in the seed dispersal direction class, but with overall no significant effect of seed weight (*p* = 0.384, χ^2^ = 0.95, Table 1).

Seed fate dispersal was then analyzed separately for gentle (α < 35°, 6 plots) and steep (35° ≤ α, 4 plots) slope gradient sites. From the seed station situated on gentle slope plots, 427 valid seed dispersal events were recorded, with 229 (53.6%) seeds transported downslope, 59 (13.8%) horizontally, and 139 (32.6%) upslope. There was a highly significant tendency for downslope seed transportation to involve longer displacement distances (*p* < 0.01, χ^2^ = 21.87). In comparison, of the 255 seed dispersals recorded on steep slopes, 123 (48.2%) seeds were transported downslope, 44 (17.3%) horizontally, and 88 (34.5%) upslope. No significant differences were observed in seed weight and dispersal distances between the groups of seeds moved in different directions (Table 2).

**TABLE 2:** Differences in seed dispersal distances in relation to slope trajectory at gentle and steep slope seed station.

Slope gradient (Group)	Number of seeds	Mean dispersal distance (m)	Downslope distance (m)	Flat‐slope distance (m)	Upslope distance (m)	Intra‐group *p*	Wald chi‐squared
Gentle Slope	427	3.58	4.17	3.32	2.72	0.000018^b^	21.87
Steep Slope	255	4.27	4.74	4.11	3.7	0.200	3.23
Inter‐group *p*‐value	—	0.013^a^	0.169	0.330	0.007^b^	—	—
Wald Chi‐squared	—	6.20	1.90	0.95	7.31	—	—

^a^ Denotes significant.

^b^ Denotes highly significant.

**FIGURE 2:**
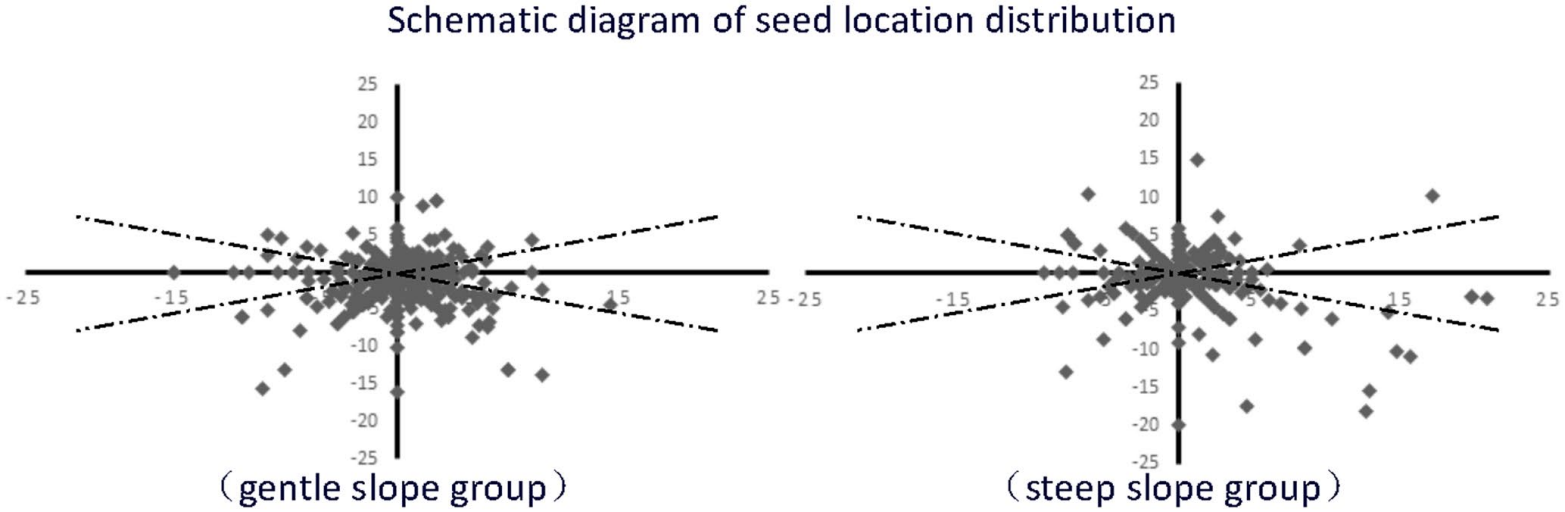
Rodent seed dispersal from release sites situated on gentle and on steep slopes.

On average, rodents dispersed seeds from release sites situated on steep slopes significantly farther in all directions than from sites on gentle slopes (*p* = 0.013, χ^2^ = 6.20) (Figure 2). This was primarily attributable to the highly significant difference in upslope seed displacement distances (*p* < 0.01, χ^2^ = 7.31). In contrast, there were no significant differences in seed displacement distances for horizontal or downslope seed displacement (Table 2).

**FIGURE 3:**
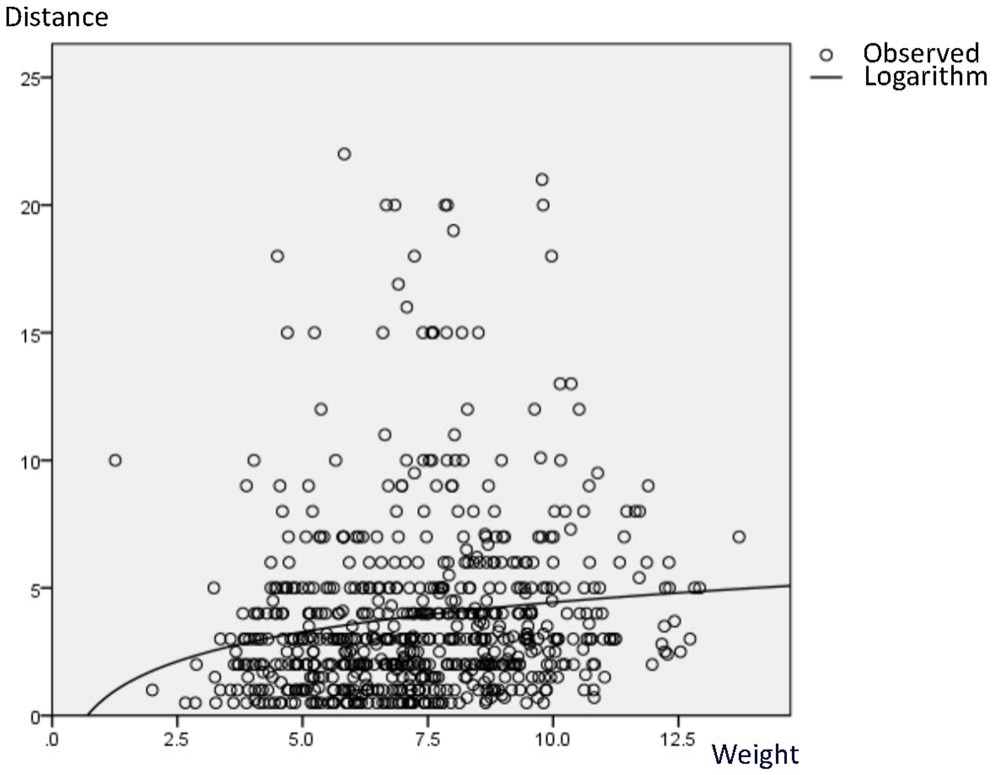
Logarithmic curve fitted for the correlation between seed weight and dispersal distance.

Using a logarithmic model, the correlation between seed weight and dispersal distance was highly significant (*F* = 14.87; *p* < 0.01). From the logarithmic curve depicting the relationship between seed weight and dispersal, heavier seeds tended to be dispersed over longer distances (Figure 3). Categorizing seeds as ‘lightweight’ (i.e., lighter than the mean, the average weight of seeds and standard deviation are:7.37 ± 2.08 g) or ‘heavyweight’ (i.e., heavier than the mean) also revealed significant differences in individual seed dispersal distances, as well as average dispersal distances per seed‐weight category (*p* < 0.01, W = 17.62), in relation to slope trajectory category (Downslope: *p* = 0.021, W = 5.32; Horizontal: *p* = 0.001, W = 10.15; Upslope: *p* = 0.039, W = 4.27; Table 3). Seed weight category did not, however, have any significant effect on the percentage of seeds dispersed in relation to slope trajectory category (*p* = 0.419, χ^2^ = 1.74). In contrast, seed weight category had a significant effect on seed fate category (*p* = 0.001, χ^2^ = 13.35; Table 4).

**TABLE 3:** Differences in seed dispersal distances in each slope direction for each seed weight category.

Seed weight class	Seed count (Seeds)	Average weight (g)	Average dispersal distance (m)	Downslope distance (m)	Horizontal distance (m)	Upslope distance (m)	*p* within group	Wald value
Lightweight seed class	351	5.66	3.30	3.91	2.53	2.76	0.002^b^	12.63
Heavyweight seed class	331	9.09	4.42	4.82	4.95	3.49	0.006^b^	10.36
*p*‐value between groups	—	0.000^b^	0.000^b^	0.021^a^	0.001^b^	0.039^a^	—	—
Wald value	—	1358.65	17.62	5.32	10.15	4.27	—	—

^a^ Denotes significant.

^b^ Denotes highly significant.

**TABLE 4:** Differences in the percentages of seeds dispersed in each slope trajectory with seed fate for each seed weight category.

Seed weight class	Seed count	Downslope percentage (%)	Horizontal percentage (%)	Upslope percentage (%)	Stored percentage (%)	Consumed percentage (%)	Dragged prercentage (%)
Lightweight seed class	351	49.9	15.7	34.5	68.7	25.6	5.7
Heavyweight seed class	331	53.5	14.5	32.0	77.9	17.5	4.5
*p*‐value between groups	—	0.419			0.001^a^		
Chi‐squared value	—	1.74			13.35		

^a^ Denotes highly significant.

Within each seed fate category, slope trajectory significantly affected dispersal distances: Dispersal distances for HAM seeds were longest for downslope trajectories (3.888 m), shorter for horizontal trajectories (3.077 m) and shortest for upslope trajectories (2.799 m). CAM seeds were dispersed significantly farther upslope and downslope than HAM seeds (Downslope: 5.827 m, Horizontal: 5.861 m, Upslope: 4.323 m; Table 5). Due to the small sample size of only 35 DAM seeds, however, further analyses were not possible for this category.

**TABLE 5:** Rodent seed dispersal distances in relation to slope direction category.

Seed fate class	Number of seeds	Average distance (m)	Downslope distance (m)	Horizontal distance (m)	Upslope distance (m)	In‐group *p*	Wald statistic
HAM	499	3.372 ± 2.79	3.888 ± 2.92	3.077 ± 3.10	2.799 ± 2.29	0.00017^b^	17.38
CAM	148	5.436 ± 4.95	5.827 ± 5.08	5.861 ± 5.99	4.323 ± 3.82	0.253	2.75
Between‐group *p*‐values	—	0.000000^b^	0.000108^b^	0.007^b^	0.003^b^	—	—
Wald statistics	—	41.58	18.27	10.06	11.38	—	—

^a^ Denotes significant.

^b^ Denotes highly significant.

There was a highly significant difference in the percentages of HAM and CAM seeds dispersed in all trajectories, especially with a significantly greater percentage of HAM seeds being dispersed upslope (Table 6).

**TABLE 6:** The percentages of seeds dispersed in each slope trajectory in relation to seed fate category.

Seed fate category	Number of seeds (*n*)	Percentage displaced downslope (%)	Percentage (%)displaced horizontally	Percentage (%) displaced upslope
HAM	499	48.7	15.4	35.9
CAM	148	58.1	15.5	26.4
Inter‐group *p*‐value	—	0.000005^a^		
Chi‐squared value	—	24.55		

^a^ Denotes highly significant.

## 4 Discussion

We found that slope topography exerted profound effects on rodent seed‐dispersal behavior, where rodents exhibited an overall bias toward foraging tactics that conserved energy. Specifically, rodents exhibited a greater tendency to displace seeds downslope and horizontally than upslope, with 1.55 times more seeds transported downhill than uphill. Furthermore, the average distance seeds were transported downhill was 1.41 times greater than uphill. This suggests that, when rodents move seeds uphill, they compensate for the additional energetic demands incurred by overcoming gravity by reducing the transport distance.

Additionally, the horizontal sector spans only 40° laterally, accounting for 11.1% of the total available area (40°/360°), whereas both uphill and downhill sectors each cover 160°, equivalent to 44.4% of the area. If the horizontal area is weighted in the same proportion as the uphill and downhill directions, the percentage of seeds dispersed horizontally (15.1% × 4 = 60.4%, combining CAM, HAM, and DAM seeds) was the highest, exceeding downhill caching by 8.8% (60.4%–51.6%) and uphill dispersal by 27.1% (60.4%–33.3%, Table 1). Considering only the final distribution of cached seeds, When only cached seeds (HAM) were considered, this disparity increased further to 12.9% compared to downhill caching (15.4% × 4%–48.7%) and 25.7% compared to uphill caching (15.4% × 4%–35.9%, Table 6). This highlights the rodent tendency to minimize particularly uphill—but also to a lesser degree downhill seed dispersal to mitigate the energetic costs associated with topographical gradients. However, due to the smaller sector area classified as “horizontal” rodents ultimately relocated a greater number of seeds downhill.

The way in which energy conservation influenced rodent foraging behavior was also sensitive to slope gradient. While rodents generally transported seeds greater distances downhill than uphill, when comparing steep with gentle slopes, this gradient effect was only significant for gentle slopes. This may be because rodents focus more on balancing and not falling when carrying seeds over the more rugged terrain on steeper slopes. Rodents can be more vulnerable to terrestrial and avian predators on rugged terrain (McDonald et al. 2020; Sullivan et al. 2001), likely favoring shorter seed translocation distances. Nevertheless, in terms of slope direction, even on steep slopes, the average distance over which rodents transported seeds downhill was still 1.28 times greater than uphill. Indeed, on steep slopes, rodents transported seeds significantly farther in all directions compared with gentle slopes (i.e., 1.19 times). Interestingly, the distance rodents transported seeds uphill was also significantly greater on steep than on gentle slopes (i.e., 1.36 times). This suggests that despite the greater energy expenditure involved in seed transport on steep slopes, greater seed translocation distances are necessitated, likely due to the scarcity of suitable seed‐caching microsites on more barren steep‐slope terrain, where rodents may need to search over a larger area to find suitable caching sites. Further investigation is needed to corroborate this inference.

While there was no significant difference in the percentage of light vs. heavy seeds transported in any direction, seed weight class was influential on transport distance. Contrary to expectations, heavier seeds were transported longer distances than lighter seeds. The percentage of heavy seeds cached also markedly exceeded the cached percentage of light seeds, while a smaller percentage of heavy seeds were consumed after movement. This suggests that transporting fewer heavier seeds presents a more efficient tactic than transporting more lighter seeds; that is, heavier seeds are more desirable and worthwhile to transport longer distances, as well as more worthwhile to cache for later consumption. This is congruent with the findings of Xiao et al. (2005), who found that in Sichuan Province dispersal distances (including mean, maximum and distribution range) of Fagaceae seeds in primary rodent caches increased significantly with seed size, and with the percentage of seeds eaten after transport. Nevertheless, for both heavy and light seeds, uphill transport distances were on average consistently shorter than downhill distances. These interactions between seed weight and transport gradient direction highlight that rodents are making complex decisions across the energy landscape in which they forage (Wilson et al. 2012), simultaneously co‐influenced by their perceived risk of predation in different habitats while engaged in seed transport, that is, the ‘landscapes of fear’ (Gallagher et al. 2017; Papastamatiou et al. 2023).

We found that, on average, seeds were transported significantly farther in all directions for immediate consumption than for caching, with the reason that consuming seed incurs no burying and concealment costs, rodents can allocate more energy to transporting seeds to a safe location for consumption (Palmer et al. 2021). As an alternative hypothesis, rodents may not be deciding a priori to transport seeds to consume them at safe sites, rather they might subsequently decide to eat the seed they are carrying if they do not encounter a suitable caching site after a certain extent of energy expenditure, that is, a ‘giving up’ tactic. In either case, the average movement distance for CAM seeds should be significantly greater than that for HAM seeds. Meanwhile, there should be no significant difference in the proportion of CAM and HAM seeds moved in different directions. But, we found that, for CAM seeds, the percentage of downhill dispersed CAM seeds was 9.4% greater than that of HAM seeds, whereas the percentage of CAM seeds transported uphill was 9.5% less than that of HAM seeds. These significant differences in the percentage downhill CAM and HAM dispersed seeds, however, would not occur if consumption was simply induced by a lack of caching sites, assuming cache site availability is equal in all slope directions. Thus, we conclude that rodents likely decide to consume or cache a seed a priori at the point of initial handling, not post hoc during moving, subsequently adjusting their movement directions and distances accordingly, where rodents can opt for downhill dispersal that consumes less energy without the need to extensively explore uphill for suitable caching sites.

In our experiments, however, it was technically challenging to discern whether the seeds were transported directly to feeding sites for immediate consumption or if they were buried at feeding sites and later excavated for consumption. For the same reason, in this study and previous studies, the movement distance was obtained by measuring the distance between the final position of the seeds and the release point, but the method could not distinguish between single or multiple transports. The “first transport” mentioned in all previous studies is actually only “the first discovered seed movement position”, and the possibility that rodents moved seeds multiple times during the recording period cannot be ruled out because the path is not tracked throughout the process. Current technologies can only reduce the probability of multiple transports by shortening the recording interval (e.g., this study uses the method of recording seed positions every day), but still cannot completely rule out secondary transports. If real‐time GPS track recording or isotope tracer technology can be used to track the movement path of seeds in the future, this problem will be properly solved. Ultimately, the *C. mollissima* seed dispersal function in this study site was performed by an assemblage of seven rodent species, which will each exhibit different dietary preferences (Verde Arregoitia and D'Elía 2020) and foraging behaviors (Sivy et al. 2011). Future research should, therefore, investigate whether species‐specific biases exist in tendencies to transport seeds up‐ or downslope. Similarly, individuals belonging to the same species typically exhibit heterogeneous scatter‐hoarding tactics, relating to their physiological state and personality (Feldman et al. 2019; Merz et al. 2023; Zwolak et al. 2020), also warranting further consideration.

## 5 Conclusion

The findings of this study indicate that differential costs due to slope and terrain can affect the behavioral strategies of rodent seed dispersal and caching behavior. When confronted with sloping terrain, rodents adjust the direction and distance of seed transport according to slope angle and seed weight. Our observations indicate that rodents likely decide seed fate (i.e., consumption vs. caching) at the initial stage of seed handling, thereby determining the direction and distance of seed dispersal. However, this inference necessitates further experimental validation.

Despite species‐based and individual‐based heterogeneity and context dependence in seed dispersal mutualisms (Brehm and Mortelliti 2022), we detected a significant bias for *C. mollissima* seeds to be dispersed downhill. While this energy conservation tactic should benefit scatter‐hoarding rodents, it opposes the directionality of seed dispersal that parent trees would benefit from in relation to rising global temperatures (Freeman et al. 2018; Liu et al. 2018); a risk known to affect chestnut species distribution in China (Xie et al. 2022). It is hoped that the clues obtained in this study will prompt researchers in the field to pay attention to this long‐neglected influencing factor (slope and terrain).

## Author Contributions

**Ning Han:** conceptualization (lead), data curation (lead), formal analysis (lead), investigation (equal), methodology (equal), resources (equal), visualization (lead), writing – original draft (lead), writing – review and editing (lead). **Jing Wang:** investigation (equal), methodology (equal), validation (supporting), visualization (supporting). **Tuo Feng:** investigation (equal), methodology (equal), validation (equal). **Jidong Zhao:** investigation (equal), methodology (equal). **Jianghong Zhang:** resources (supporting). **Xiang Hou:** investigation (equal), project administration (lead), writing – review and editing (supporting). **Gang Chang:** funding acquisition (lead), supervision (lead).

## Conflicts of Interest

The authors declare no conflicts of interest.

## Supporting information


Data S1.

## Data Availability

Data available in article Supporting Information. The data that supports the findings of this study are available in the Supporting Information of this article.

## References

[ece371388-bib-0001] Alexander, J. M. , L. Chalmandrier , J. Lenoir , et al. 2018. “Lags in the Response of Mountain Plant Communities to Climate Change.” Global Change Biology 24: 563–579. 29112781 10.1111/gcb.13976 PMC5813787

[ece371388-bib-0002] Birn‐Jeffery, A. V. , and T. E. Higham . 2014. “The Scaling of Uphill and Downhill Locomotion in Legged Animals.” Integrative and Comparative Biology 54: 1159–1172. 24733147 10.1093/icb/icu015

[ece371388-bib-0003] Bogdziewicz, M. , E. E. Crone , R. Zwolak , and M. Rees . 2019. “Do Benefits of Seed Dispersal and Caching by Scatterhoarders Outweigh the Costs of Predation? An Example With Oaks and Yellow‐Necked Mice.” Journal of Ecology 108: 1009–1018.

[ece371388-bib-0004] Brehm, A. M. , and A. Mortelliti . 2022. “Small Mammal Personalities Generate Context Dependence in the Seed Dispersal Mutualism.” Proceedings of the National Academy of Sciences of the United States of America 119: e2113870119. 35377818 10.1073/pnas.2113870119 PMC9169644

[ece371388-bib-0005] Briggs, J. S. , S. B. Vander Wall , and S. H. Jenkins . 2009. “Forest Rodents Provide Directed Dispersal of Jeffrey Pine Seeds.” Ecology 90, no. 3: 675–687. 10.1890/07-0542.1. 19341138

[ece371388-bib-0006] Cao, L. , B. Wang , C. Yan , et al. 2018. “Risk of Cache Pilferage Determines Hoarding Behavior of Rodents and Seed Fate.” Behavioral Ecology 29: 984–991.

[ece371388-bib-0007] Chang, G. , T. Z. Jin , J. F. Pei , X. N. Chen , B. Zhang , and Z. J. Shi . 2012. “Seed Dispersal of Three Sympatric Oak Species by Forest Rodents in the Qinling Mountains, Central China.” Plant Ecology 213, no. 10: 1633–1642. 10.1007/s11258-012-0118-1.

[ece371388-bib-0008] Chang, G. , and Z. Zhang . 2014. “Functional Traits Determine Formation of Mutualism and Predation Interactions in Seed‐Rodent Dispersal System of a Subtropical Forest.” Acta Oecologica 55: 43–50.

[ece371388-bib-0009] Chen, W. , Z. Zhang , C. D. Buesching , et al. 2017. “Discrimination Behavior Mediates Foraging Quality Versus Quantity Trade‐Offs: Nut Choice in Wild Rodents.” Behavioral Ecology 28: 607–616.

[ece371388-bib-0010] Chimienti, M. , J.‐P. Desforges , L. T. Beumer , J. Nabe‐Nielsen , F. M. van Beest , and N. M. Schmidt . 2020. “Energetics as Common Currency for Integrating High Resolution Activity Patterns Into Dynamic Energy Budget‐Individual Based Models.” Ecological Modelling 434: 109250.

[ece371388-bib-0011] Corlett, R. T. , and D. A. Westcott . 2013. “Will Plant Movements Keep Up With Climate Change?” Trends in Ecology & Evolution 28: 482–488. 23721732 10.1016/j.tree.2013.04.003

[ece371388-bib-0012] Cui, J. , W. Chen , C. Newman , et al. 2018. “Roads Disrupt Rodent Scatter‐Hoarding Seed‐Dispersal Services: Implications for Forest Regeneration.” Perspectives in Plant Ecology, Evolution and Systematics 34: 102–108.

[ece371388-bib-0013] Fedriani, J. M. , and A. J. Manzaneda . 2005. “Pre‐ and Postdispersal Seed Predation by Rodents: Balance of Food and Safety.” Behavioral Ecology 16: 1018–1024.

[ece371388-bib-0014] Feldman, M. , M. Ferrandiz‐Rovira , J. M. Espelta , and A. Muñoz . 2019. “Evidence of High Individual Variability in Seed Management by Scatter‐Hoarding Rodents: Does ‘Personality’ Matter?” Animal Behaviour 150: 167–174.

[ece371388-bib-0015] Freeman, B. G. , J. A. Lee‐Yaw , J. M. Sunday , and A. L. Hargreaves . 2018. “Expanding, Shifting and Shrinking: The Impact of Global Warming on Species' Elevational Distributions.” Global Ecology and Biogeography 27: 1268–1276.

[ece371388-bib-0016] Frei, E. , J. Bodin , and G.‐R. Walther . 2010. “Plant Species' Range Shifts in Mountainous Areas—All Uphill From Here?” Botanica Helvetica 120: 117–128.

[ece371388-bib-0017] Gallagher, A. J. , S. Creel , R. P. Wilson , and S. J. Cooke . 2017. “Energy Landscapes and the Landscape of Fear.” Trends in Ecology & Evolution 32: 88–96. 27814919 10.1016/j.tree.2016.10.010

[ece371388-bib-0018] González‐Varo, J. P. , J. V. López‐Bao , and J. Guitián . 2017. “Seed Dispersers Help Plants to Escape Global Warming.” Trends in Ecology & Evolution 126: 1600–1606.

[ece371388-bib-0019] Ho, X. , B. Zhang , M. Steele , et al. 2021. “Seed Traits and Rodent Community Interact to Determine Seed Fate: Evidence From Both Enclosure and Field Experiments.” Integrative Zoology 16: 939–954. 34599548 10.1111/1749-4877.12596

[ece371388-bib-0020] Kelly, A. E. , and M. L. Goulden . 2008. “Rapid Shifts in Plant Distribution With Recent Climate Change.” Proceedings of the National Academy of Sciences of the United States of America 105: 11823–11826. 18697941 10.1073/pnas.0802891105 PMC2575286

[ece371388-bib-0021] Lenoir, J. , J. C. Gegout , P. A. Marquet , P. de Ruffray , and H. Brisse . 2008. “A Significant Upward Shift in Plant Species Optimum Elevation During the 20th Century.” Science 320: 1768–1771. 18583610 10.1126/science.1156831

[ece371388-bib-0022] Lichti, N. I. , M. A. Steele , and R. K. Swihart . 2017. “Seed Fate and Decision‐Making Processes in Scatter‐Hoarding Rodents.” Biological Reviews of the Cambridge Philosophical Society 92: 474–504. 26587693 10.1111/brv.12240

[ece371388-bib-0023] Liu, B. , E. Liang , K. Liu , and J. J. Camarero . 2018. “Species‐ and Elevation‐Dependent Growth Responses to Climate Warming of Mountain Forests in the Qinling Mountains, Central China.” Forests 9, no. 5: 248. 10.3390/f9050248.

[ece371388-bib-0024] McDonald, P. J. , A. Stewart , M. A. Jensen , and H. W. McGregor . 2020. “Topographic Complexity Potentially Mediates Cat Predation Risk for a Critically Endangered Rodent.” Wildlife Research 47, no. 8: 643–648. 10.1071/WR19172.

[ece371388-bib-0025] Merz, M. R. , S. R. Boone , and A. Mortelliti . 2023. “Predation Risk and Personality Influence Seed Predation and Dispersal by a Scatter‐Hoarding Small Mammal.” Ecosphere 14, no. 1: e4377. 10.1002/ecs2.4377.

[ece371388-bib-0026] Moore, J. E. , A. B. McEuen , R. K. Swihart , T. A. Contreras , and M. A. Steele . 2007. “Determinants of Seed Removal Distance by Scatter‐Hoarding Rodents in Deciduous Forests.” Ecology 88, no. 10: 2529–2540. 10.1890/07-0247.1. 18027756

[ece371388-bib-0027] Moore, N. B. , R. B. Stephens , and R. J. Rowe . 2022. “Nutritional and Environmental Factors Influence Small Mammal Seed Selection in a Northern Temperate Forest.” Ecosphere 13: 4036.

[ece371388-bib-0028] Moore, J. E. , and R. Swihart . 2008. “Factors Affecting the Relationship Between Seed Removal and Seed Mortality.” Canadian Journal of Zoology 86, no. 5: 378–385. 10.1139/Z08-011.

[ece371388-bib-0029] Naoe, S. , I. Tayasu , Y. Sakai , et al. 2019. “Downhill Seed Dispersal by Temperate Mammals: A Potential Threat to Plant Escape From Global Warming.” Scientific Reports 9: 14932. 31624326 10.1038/s41598-019-51376-6 PMC6797773

[ece371388-bib-0030] Palmer, M. S. , C. Portales‐Reyes , C. Potter , L. D. Mech , and F. Isbell . 2021. “Behaviorally‐Mediated Trophic Cascade Attenuated by Prey Use of Risky Places at Safe Times.” Oecologia 195: 235–248. 33389153 10.1007/s00442-020-04816-4

[ece371388-bib-0031] Papastamatiou, Y. P. , B. M. Binder , K. M. Boswell , et al. 2023. “Dynamic Energy Landscapes of Predators and the Implications for Modifying Prey Risk.” Functional Ecology 38, no. 2: 284–293. 10.1111/1365-2435.14478.

[ece371388-bib-0032] Ruiz‐Labourdette, D. , D. Nogués‐Bravo , H. S. Ollero , M. F. Schmitz , and F. D. Pineda . 2011. “Forest Composition in Mediterranean Mountains Is Projected to Shift Along the Entire Elevational Gradient Under Climate Change.” Journal of Biogeography 39, no. 1: 162–176. 10.1111/j.1365-2699.2011.02592.x.

[ece371388-bib-0033] Shepard, E. L. , R. P. Wilson , W. G. Rees , E. Grundy , S. A. Lambertucci , and S. B. Vosper . 2013. “Energy Landscapes Shape Animal Movement Ecology.” American Naturalist 182, no. 3: 298–312. 10.1086/671257. 23933722

[ece371388-bib-0034] Shuai, L. , and Y. L. Song . 2011. “Foraging Behavior of the Midday Gerbil ( *Meriones meridianus* ): Combined Effects of Distance and Microhabitat.” Behavioural Processes 86: 143–148. 21070841 10.1016/j.beproc.2010.11.001

[ece371388-bib-0035] Sivy, K. J. , S. M. Ostoja , E. W. Schupp , and S. Durham . 2011. “Effects of Rodent Species, Seed Species, and Predator Cues on Seed Fate.” Acta Oecologica 37: 321–328.

[ece371388-bib-0036] Sullivan, H. L. , C. G. Curtin , C. A. Reynolds , and S. G. Cardiff . 2001. “The Effect of Topography on the Foraging Costs of Heteromyid Rodents.” Journal of Arid Environments 48: 255–266.

[ece371388-bib-0037] Vander Wall, S. B. 1990. Food Hoarding in Animals [M]. University of Chicago Press.

[ece371388-bib-0038] Verde Arregoitia, L. D. , and G. D'Elía . 2020. “Classifying Rodent Diets for Comparative Research.” Mammal Review 51: 51–65.

[ece371388-bib-0039] Wang, B. , and R. T. Corlett . 2017. “Scatter‐Hoarding Rodents Select Different Caching Habitats for Seeds With Different Traits.” Ecosphere 8: 1774.

[ece371388-bib-0040] Wang, J. , B. Zhang , N. Han , et al. 2021. “Effects of Mast Seeding and Insect Infestation on Predation and Dispersal of *Castanea mollissima* Nuts by Rodents in the Qinling Mountains of China.” Forest Ecology and Management 499: 119630.

[ece371388-bib-0041] Wang, J. , B. H. X. Zhang , X. N. Chen , et al. 2017. “Effects of Mast Seeding and Rodent Abundance on Seed Predation and Dispersal of Quercus Aliena (Fagaceae) in Qinling Mountains, Central China.” Plant Ecology 218, no. 7: 855–865. 10.1007/s11258-017-0735-9.

[ece371388-bib-0042] Wilson, R. P. , F. Quintana , and V. J. Hobson . 2012. “Construction of Energy Landscapes Can Clarify the Movement and Distribution of Foraging Animals.” Proceedings of the Biological Sciences 279: 975–980. 21900327 10.1098/rspb.2011.1544 PMC3259934

[ece371388-bib-0043] Xiao, Z. , Z. Zhang , and Y. Wang . 2004. “Dispersal and Germination of Big and Small Nuts of *Quercus serrata* in a Subtropical Broad‐Leaved Evergreen Forest.” Forest Ecology and Management 195: 141–150.

[ece371388-bib-0044] Xiao, Z. , Z. Zhang , and Y. Wang . 2005. “Effects of Seed Size on Dispersal Distance in Five Rodent‐Dispersed Fagaceous Species.” Acta Oecologica 28: 221–229.

[ece371388-bib-0045] Xie, C. , E. Tian , C. Y. Jim , D. Liu , and Z. Hu . 2022. “Effects of Climate‐Change Scenarios on the Distribution Patterns of Castanea Henryi.” Ecology and Evolution 12: e9597. 36514555 10.1002/ece3.9597 PMC9731913

[ece371388-bib-0046] Zhang, H. , Y. Chen , and Z. Zhang . 2008. “Differences of Dispersal Fitness of Large and Small Acorns of Liaodong Oak (Quercus Liaotungensis) Before and After Seed Caching by Small Rodents in a Warm Temperate Forest, China.” Forest Ecology and Management 255: 1243–1250.

[ece371388-bib-0047] Zhang, H. , C. Yan , H. Niu , H. Li , and Z. Zhang . 2022. “Masting Benefits Seedling Recruitment of Armeniaca Sibirica Through Directed Dispersal by Rodents.” Forest Ecology and Management 513: 120200.

[ece371388-bib-0048] Zwolak, R. , A. Sih , and K. Pum Lee . 2020. “Animal Personalities and Seed Dispersal: A Conceptual Review.” Functional Ecology 34: 1294–1310.

